# Sex Differences in Sand Lizard Telomere Inheritance: Paternal Epigenetic Effects Increases Telomere Heritability and Offspring Survival

**DOI:** 10.1371/journal.pone.0017473

**Published:** 2011-04-22

**Authors:** Mats Olsson, Angela Pauliny, Erik Wapstra, Tobias Uller, Tonia Schwartz, Donald Blomqvist

**Affiliations:** 1 School of Biological Sciences, University of Sydney, Sydney, New South Wales, Australia; 2 Department of Zoology, University of Gothenburg, Gothenburg, Sweden; 3 School of Zoology, University of Tasmania, Hobart, Tasmania, Australia; 4 Department of Zoology, Edward Grey Institute, University of Oxford, Oxford, United Kingdom; 5 Interdepartmental Genetics Program, Ecology, Evolution and Organismal Biology Department, Iowa State University, Ames, Iowa, United States of America; University of Turku, Finland

## Abstract

**Background:**

To date, the only estimate of the heritability of telomere length in wild populations comes from humans. Thus, there is a need for analysis of natural populations with respect to how telomeres evolve.

**Methodology/Principal Findings:**

Here, we show that telomere length is heritable in free-ranging sand lizards, *Lacerta agilis*. More importantly, heritability estimates analysed within, and contrasted between, the sexes are markedly different; son-sire heritability is much higher relative to daughter-dam heritability. We assess the effect of paternal age on Telomere Length (TL) and show that in this species, paternal age at conception is the best predictor of TL in sons. Neither paternal age *per se* at blood sampling for telomere screening, nor corresponding age in sons impact TL in sons. Processes maintaining telomere length are also associated with negative fitness effects, most notably by increasing the risk of cancer and show variation across different categories of individuals (e.g. males vs. females). We therefore tested whether TL influences offspring survival in their first year of life. Indeed such effects were present and independent of sex-biased offspring mortality and offspring malformations.

**Conclusions/Significance:**

TL show differences in sex-specific heritability with implications for differences between the sexes with respect to ongoing telomere selection. Paternal age influences the length of telomeres in sons and longer telomeres enhance offspring survival.

## Introduction

Telomeres are thought to have several vital functions in addition to protection of the chromosome ends, such as providing a mechanism for distinguishing between real chromosome ends and breaks that need repairing, having a role in alignment and segregation of chromosomes during meiosis, modifying gene expression, and contributing to stress resistance in cells and tissue [1]–[3]. We show elsewhere that in sand lizards (*Lacerta agilis*), there are sex-specific effects relating to telomere length (TL), such as stress-tolerance from predation [4] and positive selection in the wild relating to increased lifespan and lifetime reproductive success (stronger in females; 4; oral report at the Swedish Telomere and Telomerase Network Annual Meeting, Sven Lovén Centre, May 2010; Olsson *et al.* submitted). However, the genetic architecture and epigenetic aspects of the underlying processes determining telomere length have not been addressed, which makes it difficult to assess the evolutionary implications of selection patterns. Most notably, evolutionary responses to selection depends on heritability for telomere length (since selection depletes genetic variance and alters allele frequencies) [5]–[6] but evidence from natural populations is restricted to human twin studies [7]. Furthermore, intergenerational relationships of telomere length can be complex (heretoforth TL; our own work results in lengths of Terminal Restriction Fragments, TRF, and are referred to as such). Human studies have demonstrated that paternal age at conception influences TL in infants (human germ line cells have telomerase activity throughout life and telomeres therefore become longer with male age when diploid stem cells differentiate into haploid spermatozoa [8], see also [9] on paternal inheritance of TL). Thus, depending on an individual's stress tolerance and ability to withstand genetic erosion, the TL could be epigenetically adjusted and influence offspring fitness accordingly through phenotype-specific transmission of TL (as opposed to strict inheritance of germ-lined derived genes; [10]–[11]). However, such transgenerational effects, and their fitness consequences, are yet to be explored in non-human animal systems.

Also the sex determination system may affect telomere inheritance by generating asymmetries between father-offspring and mother-offspring estimates of heritability [5]–[6]. However, current evidence is highly contradictory; Nordfjäll *et al.* showed strong heritability between fathers and sons and daughters, but no such effects between mothers and offspring [7]. Nawrot et al., however, concluded that the lack of heritability between fathers and sons in their data, but robust correlations between fathers and daughters and mothers and both-sexed children, suggests X-linked inheritance [8]. Work of others also showed that telomeres on the inactive X chromosome suffer from a faster attrition than on the X that has not been silenced [9].

In sand lizards, *Lacerta agilis*, females are the heterogametic sex with a genetic sex determination system of ZW/ZZ [12], with no information available on the gene content of the sex chromosomes, except that any telomere inheritance will obviously not be X-linked. Quantitative genetics theory predicts lower heritabilities for males and females to reflect past history of selection if it has been strong enough to erode additive genetic variance with less current heritability for the sex having been under the strongest selection. In other words, there should be higher heritability for the sex in which telomere length is less strongly impacting fitness [6]. Because of the difference in selection pressures in males and females, with stronger fitness benefits in females than males, and the potential importance of (sex-specific) intergenerational effects on telomere length, we designed a study allowing us to estimate sex-specific heritability of TL in a natural sand lizard population.

Age has a complex effect on TL in sand lizards. In females, there is a weak positive correlation between TL and age, which is likely to be caused by longer lifespan in females with longer telomeres rather than elongation of TLs through life; the latter interpretation stems from a repeat sampling of nine females which showed no change in TL between sampling events [4]. In males, there was a trend (*P* = 0.074) for a shortening of TL with age [4] and a closer examination showed that when the male population was truncated, males older than ca 3 years had shortening telomeres (Olsson unpublished). Thus, it is not obvious whether age should be controlled for in our analyses, in particular with regards to comparisons between the sexes with respect to TL heritabilities. We therefore tested for heritability differences between the sexes in two ways, first using raw data, and then using residuals from a TL-age (years) regression.

 Given the evidence of telomere-related viability from humans and laboratory animals, an outstanding question in field biology is still whether TL is genetically and epigenetically inherited in the wild, and whether offspring survival is influenced by parental TL characteristics. Furthermore, ‘No other chromosome structure (but telomeres) has been linked to major human health issues as tightly as telomeres, and in particular their length, to the point that TL has become an obliged biomarker for anyone analyzing the impact of any factor (either environmental or genetic) into human fitness, *a forteriori* in aged populations’ [13]. Given this, a natural step in evolutionary ecology has been to assess links to lifespan and life history evolution (summarised in [14]) and covariation between age and telomere dynamics [15], [16]. However, a large body of evidence from the biomedical literature tells of the complexities of telomere regulation, elevated attrition from oxidative stress [3], and epigenetic master regulators of telomere dynamics such as histone and DNA methyltransferases [10]. Thus, a plethora of factors could influence also offspring fitness via epigenetic pathways.

 In this study, we (i) addressed whether TL in sand lizards show sex-related heritability, (ii) and if TL declines with age in males. (iii) We then tested if TL of fathers were inherited to their sons and whether there was evidence that paternal age had a direct effect on TL of sons (indicating transgenerational effects). (iv) Using a data set on lifetime reproductive success of males with known TL, we address whether TL was significantly associated with offspring survival.

## Materials and Methods

This work has been approved by the Ethics committees at the University of Gothenburg, Sweden, and the University of Wollongong, Australia (Mats Olsson's employer at the time), permits AE04/03–05.

We studied sexual dimorphism in telomere heritability in free-ranging male and female sand lizards on the Swedish west coast, a population we have monitored since 1984. Eighty males (40 son-sire pairs) and 110 females (55 daughter-dam pairs) of known age at blood sampling (from hatching data) were sub-sampled from our data set on 3,968 microsatellite genotyped males and females using up to 21 microsatellites [17].

### Lizards and site location

Sand lizards (*Lacerta agilis*) are sexually dimorphic, small (to 20 g), ground-dwelling lizards. Females at this site lay one clutch of approximately 4–15 eggs per year and more polyandrous females have a reduced risk of having developmentally compromised (‘malformed’) offspring [18]. The population at our main study site (Asketunnan, ∼N57°22′ E11°58′) has been studied for over two decades and detailed descriptions of field techniques can be found elsewhere [18]. In brief, individually marked lizards were monitored during each reproductive season throughout their lives. Females were brought into the laboratory a week before egg-laying. Eggs were harvested within hours of laying and incubated at 25°C [18]. The lizards were weighed and measured (snout to vent, mm), and had a ca 50 µl blood sample taken [18] for DNA extractions. The choice of tissue was based on the following three reasons: (i) Haussman and Vleck [19] suggested blood as an excellent candidate tissue for telomere analysis, since telomeres in blood cells may shorten at a greater rate than in other tissue, (ii) destructive sampling, such as necessary for comparison of telomere traits against germ line tissue [19] is not possible in longitudinal studies requiring uncompromised viability and longevity, and (iii) blood sampling is quick and easy under field conditions [18]. At hatching, male hemipenes can be gently everted and this was used to sex all offspring [20], and reconfirmed to have near 100 percent repeatability [18]. Malformations were scored by eye (e.g. missing extremities, warped spines, asymmetric jaws; 1 = malformed, 0 = not malformed). A ca 10 µl blood sample was taken from *v. angularis* (in the corner of the mouth) of hatchlings (for more detailed description, see [18]). For a subset of males the exact age of fathers at the conception of their sons (*N* = 12) was known from hatching records of both sires and sons. A 0.6 km corridor surrounding the study site, representing more than sixty average female home ranges wide, was routinely monitored for migration and has been verified sufficient to remove any bias of estimation of survivorship [18].

### Molecular genetic analysis

**Figure 1:**
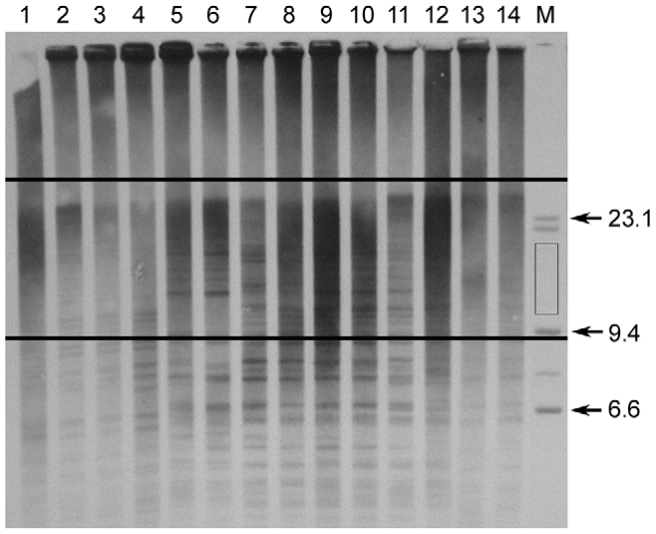
Telomeric profiles of sand lizards. Telomere fragments (lanes 1–14) were size-separated on a non-denaturing agarose gel together with two size standards (lane M, second standard not shown) and detected using chemiluminescence. The mean length of TRFs was estimated within the outlined window using the size standards (fragment sizes shown in kilobases, see Methods for details). The rectangle specifies the area used for background subtraction.

Telomere restriction fragments (TRFs) were prepared as previously described [4]. In brief, samples were stored at−20°C in either Tris-EDTA buffer or ethanol until high molecular weight genomic DNA was carefully prepared from whole blood using proteinase K digestion at 37°C and standard phenol/chloroform extraction [21]. We digested 5 µg of genomic DNA with *Hae*III and size-separated TRFs on a 0.6% non-denaturing agarose gel for 23 hours at 2 V/cm using constant-field gel electrophoresis. Samples were randomly loaded on gels in order to avoid any bias due to loading order, and two λ/*Hind*III size markers placed at each end (to assess uniformity of DNA migration across the gel). After standard Southern blotting [22], TRFs were hybridized to an alkaline phosphatase-linked telomere-specific probe and detected by chemiluminescence using CDP-Star (1,2-dioxetane) as a substrate (AlkPhos labeling and detection kit, GE Healthcare); see [4], [5] for further details on similar non-radioactive labelling techniques. Until recently, probes have typically been labelled with the radioactive isotope ^32^P. However, non-radioactive labelling systems, such as the one described above, were shown to be 500 times more sensitive than the traditional method [23], [24]. Digitalized signals were analyzed as previously described [21]. The upper limit of the window was set to include the distinct start of the smear, corresponding to the longest TRFs at approximately 30 kb. The lower limit was selected to coincide with the size standard fragment that marked the approximate border above which a clear signal was present (9 kb) and to avoid signals from telomere-like interstitial regions (approximately below 10 kb; see [25]. The analysis of TRFs with TELOMETRIC complied with all relevant conditions (e.g. type of electrophoresis and window of analysis) for which the program has been validated ([26]; M. Ochs, pers. comm.). A representative picture of telomeric profiles of sand lizards and the corresponding statistical data output obtained with TELOMETRIC are shown in Figure 1 and Table 1. While this MS was in review, a large selection analysis of ours submitted elsewhere and based on Telometric-derived data was queried since Telometric (due to its interpolation algorithm) may inflate the length of assessed telomeres (Haussman, Salomons & Verhulst, in press). For that study, we therefore re-estimated TRF lengths using the more conventional imaging software ImageJ in two ways, for the same analysis window as we used for Telometric (9–30 kb), and for the entire length of the gel lane produced per individual. As predicted by Haussman et al. (in press), both our ImageJ assessments of TRF length were shorter in length compared to our Telometric data. The analysis window for which Telometric gave an average TRF length of 18.20 kb±0.83, SD, ImageJ analysis resulted in a TRF length of 14.84 kb±1.40, SD, whereas the corresponding results analysed over the full gel lanes gave an average TRF length of 10.62 kb±1.51, SD, for both sexes combined (*n* = 128). The ImageJ estimates were strongly correlated with each other (*r* = 0.76, *P*<0.0001), and both these estimates were significantly correlated with our Telometric data (*r = *0.55, *P*<0.0001 and *r* = 0.42, *P*<0.0001, for the same versus full length estimates, respectively). These analyses showed that there was more difference within ImageJ data than between ImageJ and Telometric in terms of identifying selection on TL. In our experience, we therefore agree with Haussman et al. (In press) that Telometric creates a longer TRF estimate than does ImageJ, but this appears not unlike a difference caused by a simple scaling factor and only has importance when the exact length of telomeres are considered (e.g., in comparative analysis across taxa). Thus, it is important to note that this is in principle no different to an analysis based on qPCR or similar method aimed at identifying relative rather than absolute differences among individuals). Furthermore, Telometric does have its advantages compared to ImageJ, such as an in-built control of background ‘noise’ (the greyscale intensity of the background is automatically deducted from the lane intensity), which is consistently applied whereas this is left to the researcher to apply in ImageJ (which is far from straightforward to do consistently). Since all our analyses here are based on analyses of interindividual, relative differences in TL and its impact on inheritance and fitness factors, we have therefore stayed with reporting only on Telometric data for reasons of repetitiveness and space use.

**Table 1:** Data output corresponding to samples (lane 1–14) shown in Supplement 1.

Lane	Mean (kb)	Median (kb)	Mode (kb)	Variance	SIR (kb)
1	18.45	17.77	15.45	34.46	6.76
2	19.4	19.59	23.85	32.68	7.36
3	19.22	19.23	10.15	37.94	6.92
4	17.4	16.41	9.85	36.94	5.95
5	18.14	17.56	8.85	34.58	6.49
6	18.62	18.26	12.35	32.08	6.81
7	17.59	16.74	8.85	33.16	6.29
8	17.59	16.78	8.85	35.82	6.12
9	17.3	16.3	8.85	35	5.99
10	17.08	16.21	8.85	32.53	6.02
11	18.57	18.08	10.85	37.76	6.44
12	18.25	17.6	8.85	36.81	6.46
13	17.61	16.87	9.01	33.5	6.23
14	17.86	17.27	8.91	33.38	6.28

Statistical measurements produced by TELOMETRIC include the mean, median, mode, variance, and semi-interquartile range (SIR). The latter gives the spread in kilobases (kb) spanning the 25th to 75th percentiles (divided by 2).

### Data analysis

Our analysis is based on mean offspring survival (response variable) from sires with known lifetime reproductive success, with DNA available from prior sampling, and haphazardly collected for the subsequent TL screening (in our case using TRFs). Analyses reported on in the current manuscript are based on adults of known date of birth and year of death and with all their offspring assigned through molecular microsatellite assignment (see further below). The rationale for incorporating offspring sex in the analysis is that we know that there are sex-specific effects on risk of being malformed, and concomitant risk of dying in this species [27], [28]. Offspring mean sex ratio is therefore included to make TL effects on offspring survival independent of previously published results but will not be further elaborated on in this report. The rationale for using mean offspring values was that paternal TL was only sampled once (and made age-independent using residuals from a TRF-age at sampling regression). Using repeat observations of male TRFs sampled once only may therefore lead to pseuoreplication if offspring are used as independent observations (e.g. in a mixed model survival analysis with father identity as a random factor).

## Results

### Telomere heritability

**Figure 2:**
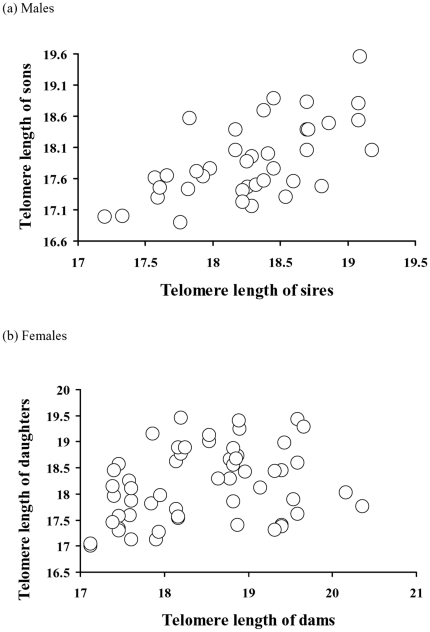
Heritabilities of telomere length (kb) in sand lizards. In (a) males, the heritability between sons and sires was 1.23 (from a regression coefficient of 0.62±0.11, SE, n = 40). In (b) females, the heritability between dams and daughters was 0.52 (from a regression coefficient of 0.26±0.11, n = 55).

We calculated heritability of telomere length in daughters regressed on maternal TRF (n = 55) and adult sons regressed on sire TRF (n = 40). In the females, there was significant heritability (β = 0.26±0.11, i.e. h^2^ = 0.52, R^2^ = 0.094, F_1, 54_ = 5.57, p = 0.022), whereas for males the corresponding analysis not only showed significant heritability but four times greater proportion of explained variance compared to females (R^2^ = 0.44) and a heritability greater than one (β = 0.62±0.11, i.e. h^2^ = 1.23, F_1, 39_ = 30.34, p<0.0001). To assess whether these heritabilities were indeed different we also performed a homogeneity of slopes test, which demonstrated significant effects of both main effects (TRF_parents_, F = 26.55, p<0.0001; Sex, F = 4.84, p = 0.030, dfs = 1) and their interaction (Parental TRF x Sex, F = 4.51, p = 0.036; Fig. 2 a, b). Thus, this interaction effect demonstrates a sex-difference in parental-offspring TRF relationship and this result was virtually identical when using residuals from the TRF-age regression rather than raw data (F = 4.66, p = 0.034).

For a subsample of sons we also had the TRFs of both sire and dam. We therefore ran a multiple regression analysis with the son TRF as response variable, and those of sires and dams as predictors. In this analysis the paternal TRF effect was significant whereas the dam effect was not (Model F_2, 17_ = 4.6, p = 0.027; R^2^ = 0.38; TRF_dam_, F = 0.0, p = 0.97, df = 1; TRF_sire_, F = 9.3, p = 0.008, df = 1). The corresponding data for daughters was not available to us.

### Paternal and epigenetic effects of age

**Figure 3:**
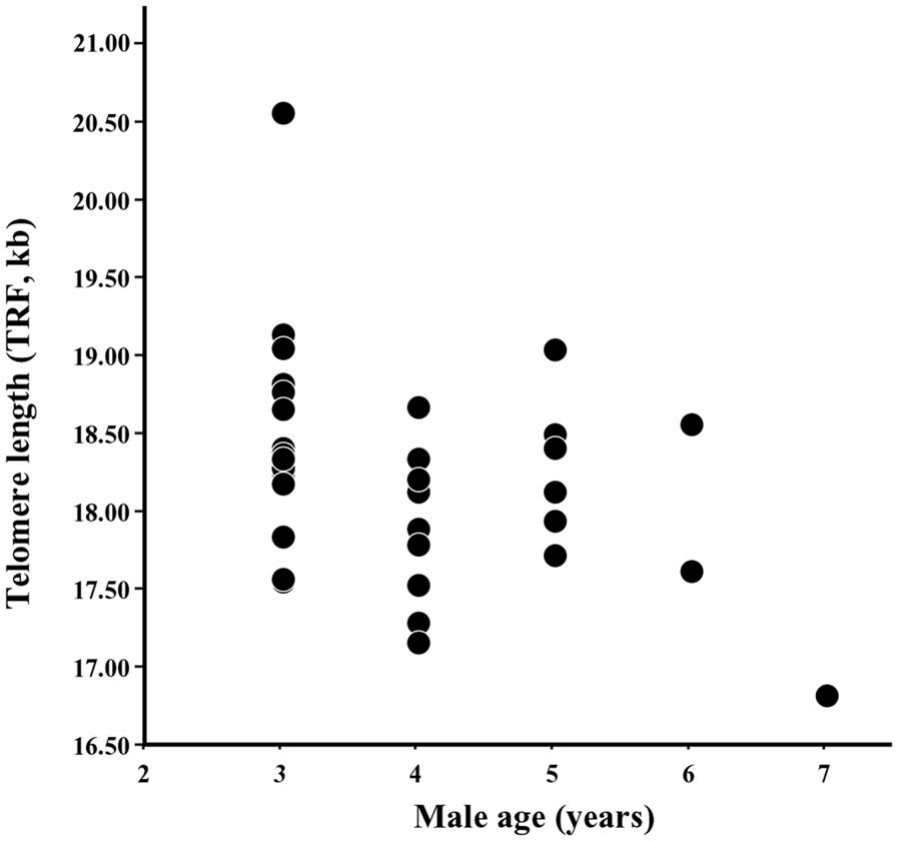
Relationship showing a negative relationship between TL and age in sire males (r_s_ = −0.34, p = 0.03, n = 40).

**Figure 4:**
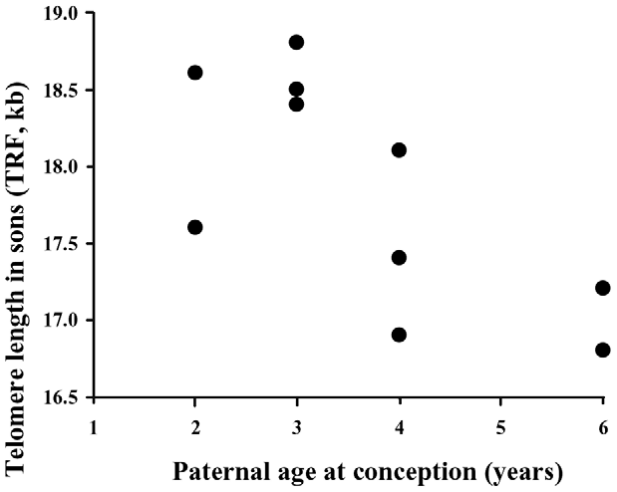
Relationship between male age at conception and the corresponding TL of their sons. The lowest TRF observation for age 2 and the highest for age 3are duplicate observations (n = 12).

The average age of sampled sires was 4.5 years±1.6 SE (range 3–7) and of sons 3.2 years±0.4 (range 2–6) and the age of sires and sons were uncorrelated (r_s_ = −0.23, p = 0.16). In sires, there was a negative relationship between age and TRF (Fig. 3, r_s_ = −0.34, p = 0.03, n = 40), but this was not true in sons (r_s_ = −0.03, p = 0.85, n = 40). Thus, TRF erosion seems to be age-dependent and only evident in older males. Furthermore, paternal age at conception was significantly negatively correlated with the TRF length of their sons (Fig. 4, r_s_ = −0.59, p = 0.041, n = 12). Note that a negative correlation between paternal age and TRF of sons strengthens their TRF similarity if there is vertical TRF transfer via sperm.

**Figure 5:**
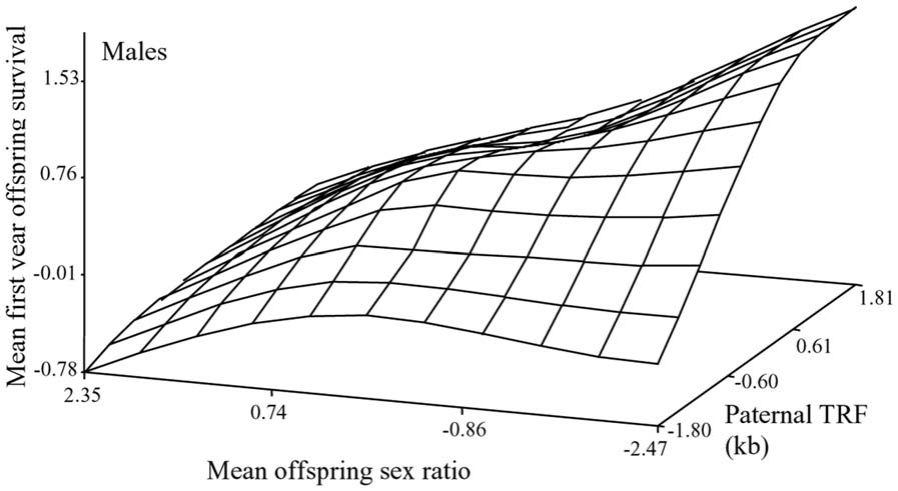
Response surface plot illustrating the effects on mean offspring survival of paternal TL and the mean sex ratio of their offspring sired throughout their life time (0 = all daughters). The regression analysis is given in full in Table 2.

Since all TRF estimates in this study come from adult males of different ages, and old males suffer from TRF erosion, we tested whether age-specific male TRF length (residuals from a TRF-age regression) affected the mean survival of all offspring a male produced through his life (survived = 1, not survived = 0). This showed that residual TRF length of sires significantly increased the mean recapture rate of the offspring (p = 0.013; see Table 2 for all test statistics), independently of significant effects also of mean offspring sex ratio (p = 0.009; Table 2), and mean frequency of malformations (p<0.0001; Table 2). This is illustrated in our response-surface plot (Fig. 5), which shows how mean offspring survival increases approximately linearly with paternal TRF.

**Table 2:** Results from a multiple regression analysis examining the effects of age-independent telomere length, mean offspring sex ratio through a sire's lifetime, and malformation frequency on average of a male's offspring sired through his lifetime.

Traits	df	SS	F	P
TRF (residual from age)	1	2025	6055	0.013
Mean offspring ratio	1	2.55	7.40	0.009
Mean frequency of malformations	1	6.16	17.9	<0.0001

Model statistics:*F*_3, 58_ = 8.46, *P*<0.0001, *R* ^2^ = 0.30.

## Discussion

Estimating heritability of telomere length in natural populations from individuals of known age and sex is important for addressing its evolutionary potential and may allow an assessment of whether, for example, intergenerational effects due to reduction in TL in sperm in older males and the sex-determining system contributes to this potential. Our data on sand lizards, a species where TL is under stronger positive selection in females than in males, show that TL (estimated as TRF) is heritable in both sexes with higher heritability for son-father than for daughter-mother comparisons.

Sexual differences in telomere length and attrition has been suggested to contribute to sex-specific disease and mortality patterns in humans ([29]–[31]; women typically have longer telomeres and are longer-lived). However, despite the overwhelming current interest in telomere biology (e.g. the awarding of the Nobel Price 2009 in medicine and physiology, [32]), we know virtually nothing about the quantitative genetics processes in the wild relating to sex differences in telomere dynamics, such as their heritabilities. That is, the actual evolutionary processes that contribute to the sex-specific patterns we observe are usually attempted to be indirectly explained using clinical research rather than by the more appropriate quantitative genetics analyses designed for this purpose.

How robust are our analyses and is there any risk that our interpretations result from biases in sampling of males and females? We doubt this since males and females are identically treated in terms of field and lab protocols in our study. Male heritabilities higher than one are notable since classic quantitative genetics predicts a maximum heritability of one. However, a number of processes can lead to this result, for example, a genetic relatedness greater than theoretically expected (which could be the result of intergenerational transfer of TL between fathers and sons). Inbreeding in small populations can also contribute to such bias [5]. Sand lizards show relatively low genetic variation for a diploid vertebrate [33], [34] but for this to cause a sex-bias on TL there needs to be sex-specific effects of inbreeding on telomere regulation and no such effects have been studied to date. Furthermore, in humans, Y-linked inheritance may cause paternal half-sibs to have correlations equal in magnitude to full sibs with a greater heritability for half- than full sibs and larger than one in magnitude [5]. However, this would suggest a reversed scenario in sand lizards to what we have empirically observed, with higher heritabilities in females, since females are the heterogematic sex (and this would suggest W-linked inheritance). Lastly, our analyses are based on offspring-parent regressions, which are known to result in higher heritabilities on average than more recently adopted animal models [35], largely due to higher precision in repeat measurements of traits adopted in animal models. However, in our models sample sizes are approximately equal between males and females and there is no difference in how data has been sampled.

We thus remark on the pronounced differences in heritability and unexplained variance in the son-sire versus daughter-dam analyses. Both sexes show significant heritability with identical protocols from field studies, to blood sampling, to molecular genetic analyses. However, much higher heritability in males agrees with several scenarios; females have been under stronger selection for TL via its effects on lifetime reproductive success through the evolutionary history of this population and this may have led to the depletion of genetic variation for telomerase regulation (e.g., via genes regulating TERC, the template RNA, or TERT, the catalytic reverse transcriptase; [36]) relative to any sex-specific mutational processes that maintain this genetic variation. Alternatively, some, as yet, unidentified mechanism of inheritance of TL between fathers and sons may result in the observed heritability. One such possibility would be if genetic determinants of TL are Z-linked and silenced differently depending on paternal or maternal inheritance; such Z silencing through cytosin methylation has recently been verified in birds, although less pronounced than the corresponding X silencing in mammals [37]–[39]. Alternatively, Z-linkage would also mean that any such telomerase-regulating genes would spend two thirds of their lives being under selection for a more male-beneficial genotype (since females are ZW heterozygotes). One such example could be if haploinsufficiency pathology is better buffered in males than females [36], [39].

An overlooked aspect of TL biology in free-ranging animals is thus probably inter-generational transfer of TL via sperm and its potentially associated transgenerational fitness effects set by the adjustment of paternal TL by genetic erosion from age and stressors prior to conception. Such paternal sperm TL effects are believed to explain why the relative TL among human newborns remains the same throughout life and, hence, seems to be determined at the stage of fertilization [40]. In our sample of male sand lizards, TL was highly heritable but decreased with age and older males sired offspring with shorter telomeres. Since offspring were sampled before the onset of telomere shortening (around three years of age), this suggests that telomere shortening with age in blood cells may also reflect TL in sperm and, thus, be epigenetically inherited. Furthermore, the patterns that we describe in the present study may further increase in precision and resolution if TL were used directly from spermatozoa in these analyses. It is worth noting, however, that erythrocyte TL was also used in the human studies for which positive correlations between sire age and offspring TL were described [9]. Our work also suggests that telomerase activity in reptilian spermatozoa may differ to mammalian (where it prolongs sperm TL through life; [9]). In sand lizards, the action of telomerase throughout life, and to what degree it varies in activity through different tissues, is unknown. The only Squamate reptile studied in this regard to the best of our knowledge is the python *Liasis fuscus* [41], which interestingly has TL that increase in their first year of life but then decrease later in life. Qualitatively, this suggests some similarity in pattern to sand lizards to the extent that TL decrease significantly only later in life.

In the last decade or so, demographers and other evolutionary ecologists have addressed to what degree TL can be predicted to covary with lifespan across species and clades. For instance, work on long- and short-lived sea urchins (*Strongylocentrotus franciscanus, Lytechinus variegatus*) has shown that telomere fragments are maintained throughout life, with maintained telomerase activity throughout developmental stages and in adult tissue without causing neoplastic transformation of tissue [42]. However, telomeres can have profound effects on a number of cytogenetic functions both late and early in life [2], [6], which may make it hard to know exactly if and when TL curtails fitness, and whether this effect is independent of senescence [43], [44]. For example, it has been suggested that ‘embryo senescence’ is a result of telomere shortening in response to oxidative stress [45]. This could be explained by a reduction in catalase activity in response to telomerase deficiency (in cell culture; [46]), but to what extent this applies in whole-organism biology remains unexplored. If TL is indeed an important component of phenotypic quality, any genetic and environmental influences on TL at conception or the rate of attrition during ontogeny would have consequences for individual fitness. Furthermore, telomere dynamics and its potential role as a biomarker of ageing has received considerably mixed reviews in the biomedical literature, with twin studies showing that TL does indeed predict survival in advanced age groups, largely independently of other genetic influences [47], while other both cross-sectional and longitudinal studies have shown the opposite [48]. A large body of biomedical literature and reviews outlines aspects that contributes to telomere dynamics (e.g., levels of oxidative stress, oestradiol, chronic stress, epigenetic regulation, and length-dependent telomere attrition [1], [10], [14]). We point out an additional factor here, namely paternal age at conception, which may reflect telomere change in sperm with age (in this case shortening).

What may explain paternal TL effects on offspring survival-pathology? There is a plethora of reviews on human telomere biomedicine, and case studies with broad coverage of the literature on disease related to TL, typically focusing on pathology, such as cancer in older age groups [49], [50]. In our very broad literature survey for this paper, we reviewed more than 1,000 journal hits on a wide variety of key words (including juvenile, offspring, hatchling and young in combination with telomeres) with extremely few hits depicting pathology at early, post-partum life stages (most likely because younger age groups have been relatively unstudied). One paper reports increased risk of embryo fragmentation in IVF-treated women in response to telomere shortening (on a theoretical background of work on mice that demonstrated impaired chiasmata and embryonic cell cycles, which promoted apoptosis; [51]). Another report demonstrated reduced TL in the kidneys (and liver) as a response to catch-up growth in male rats and a reduced life span-but with the reduction in life span taking place relatively late in life (i.e., not being manifested as an increase in juvenile mortality due to kidney and liver failure [45]). Thus, we conclude that there is no, or very little, work that evaluates the effects of TL and attrition during early post-partum life-in free-ranging populations there is none.

In summary, we show that heritability estimates of telomere inheritance are much higher when estimated from sires to sons than from dams to daughters. In a subsample in which telomere length for both parents are known and contrasted as predictors of telomere length in sons, only the sire was significant. There are several alternative explanations for this, including ongoing selection that may deplete genetic variation of telomere regulation more in females than males. However, the very high heritability between sons and sires may suggest a more direct, transgenerational link of TL determination, than mere sex-specific depletion of genetic variance for TL regulation. Our work suggests that paternal TL may be epigenetically inherited from fathers to sons (daughters not tested here), and that this influences their chances of survival early in life. Our data encourages biologists across disciplines to take a broader approach to telomere biology than the current focus on telomere's role in ageing, and also explore developmental and sex-specific effects of telomeres in organismic evolution. This has exciting implications for future studies of evolutionary genetics and potential sexual conflict of TL regulation, perhaps in particular in animals with ZW sex determination, such as birds and Squamate reptiles (lizards and snakes).
